# Supplementary material for: Metabolic balancing by miR-276 shapes the mosquito reproductive cycle and Plasmodium falciparum development
Source: Nat Commun. 2019 Dec 10;10:5634. doi: 10.1038/s41467-019-13627-y (PMC6904670; doi:10.1038/s41467-019-13627-y)
Supplement: Supplementary file 1 — Supplementary Information [file 41467_2019_13627_MOESM1_ESM.pdf]

Metabolic balancing by miR-276 shapes the mosquito reproductive cycle and *Plasmodium falciparum* development

Lampe *et al.*

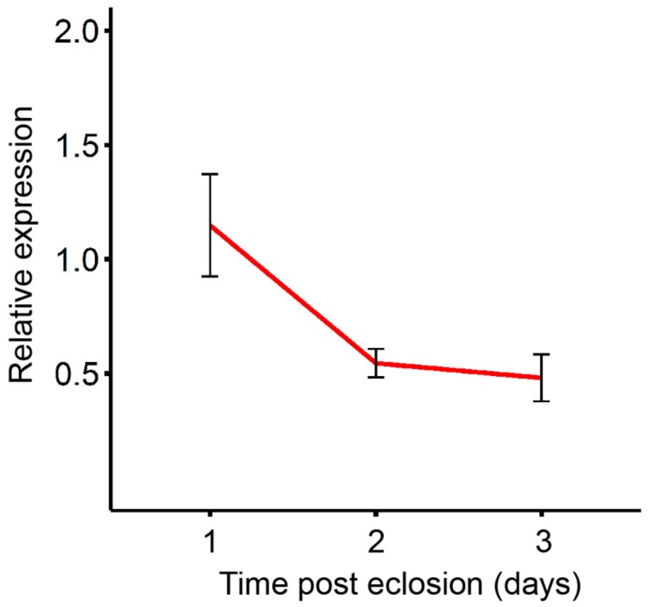

Supplementary Figure 1. Expression of miR-276 in the fat body of female mosquitoes post eclosion. miR-276 expression in the fat body of mosquito females (n=10) at day 1, 2 and 3 post eclosion. Expression levels were normalized using the ribosomal protein *RPS7* gene. Data shown as mean +/- SEM (N=3) (n = number of mosquitoes pooled for each independent experiment; N = number of independent experiments).

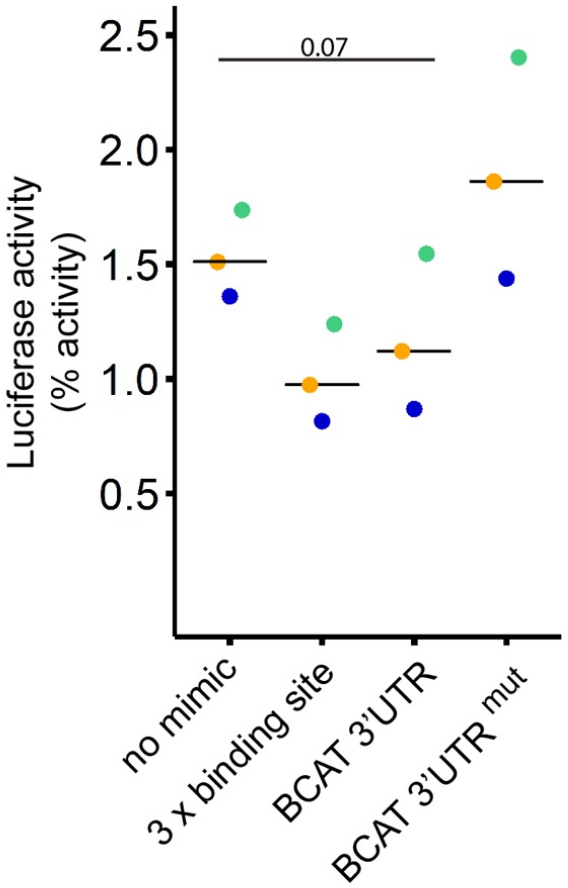

Supplementary Figure 2. miR-276 directly targets the 3′-UTR of *BCAT*. Dual luciferase reporter assay *in vitro* using *Drosophila* S2 cells. The reporter plasmid containing three miR-276 binding sites (3 x binding site) served as a positive control. Reporter activity was induced in the absence of miR-276 mimic construct (no mimic) by the endogenous to S2 cells activity of *Drosophila* miR-276. Significant differences examined by one-way ANOVA (n=3, N=3) are shown (n = number of technical replicates within the experiment; N = number of independent experiments).

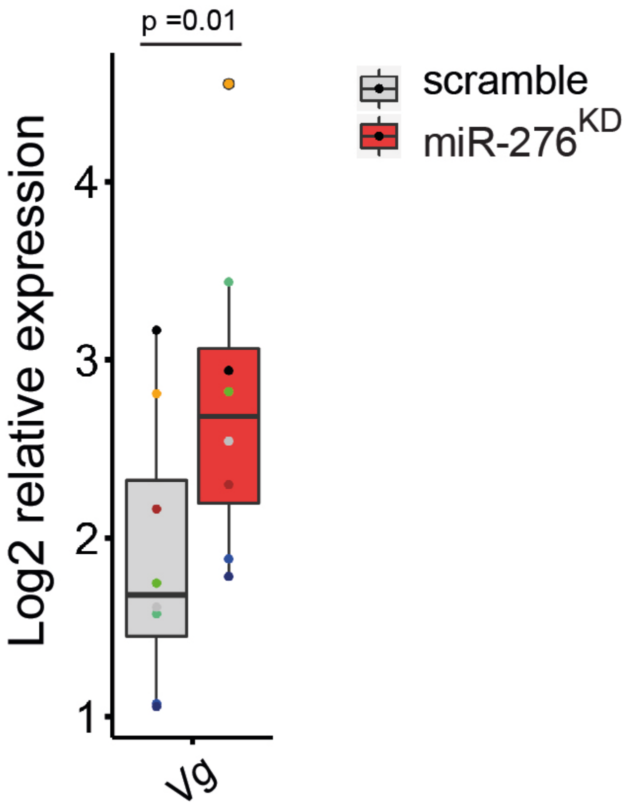

Supplementary Figure 3. Knockdown of miR-276 increases *Vitellogenin (Vg)* expression at 38 h post blood feeding. Log_2-transformed expression of the yolk protein *Vitellogenin* (Vg) at 38 h post blood feeding in the fat body of mosquitoes (n=5, N=7) injected with anti-miR-276 (miR-276, red) or scrambled antagomir (scramble, grey). Expression levels were normalized using the ribosomal protein *RPS7* gene. Boxplots show the median with first and third quartile, whiskers depict the min and max. Statistical significance was examined by t-test and significant differences are shown by the *p*-value above the horizontal line.

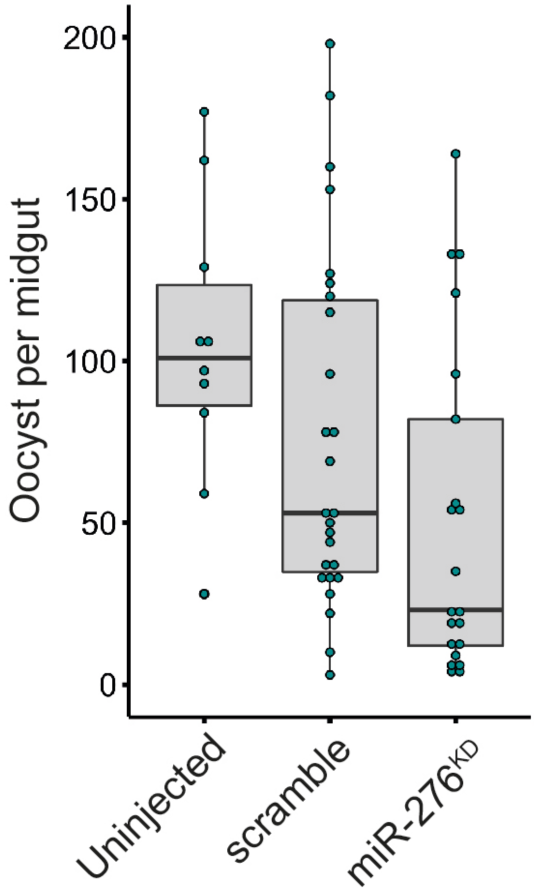

Supplementary Figure 4. Injection of antagomirs results in reduced parasite development. Uninjected females and females injected with miR-276 (miR-276^{KD}) or control (scramble) antagomir were infected with *P. falciparum*. The number of developed oocysts was counted 11 days post infection (N = 1; n > 10).

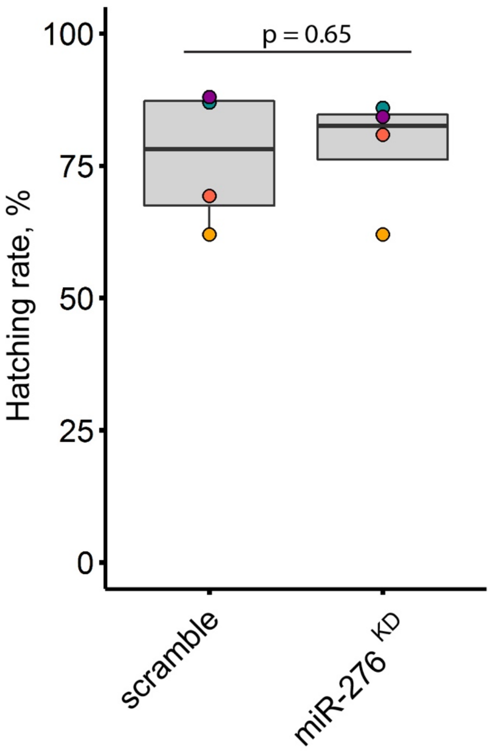

Supplementary Figure 5. miR-276 silencing does not affect larval hatching rates. Larval hatching rates of eggs laid by females injected with anti-miR-276 (miR-276^{KD}) or scrambled antagomir (scramble). Boxplots show the median with first and third quartile, whiskers depict min and max values. Each dot color represents one independent experiment with at least 100 eggs (N = 4, N = number of independent experiments).

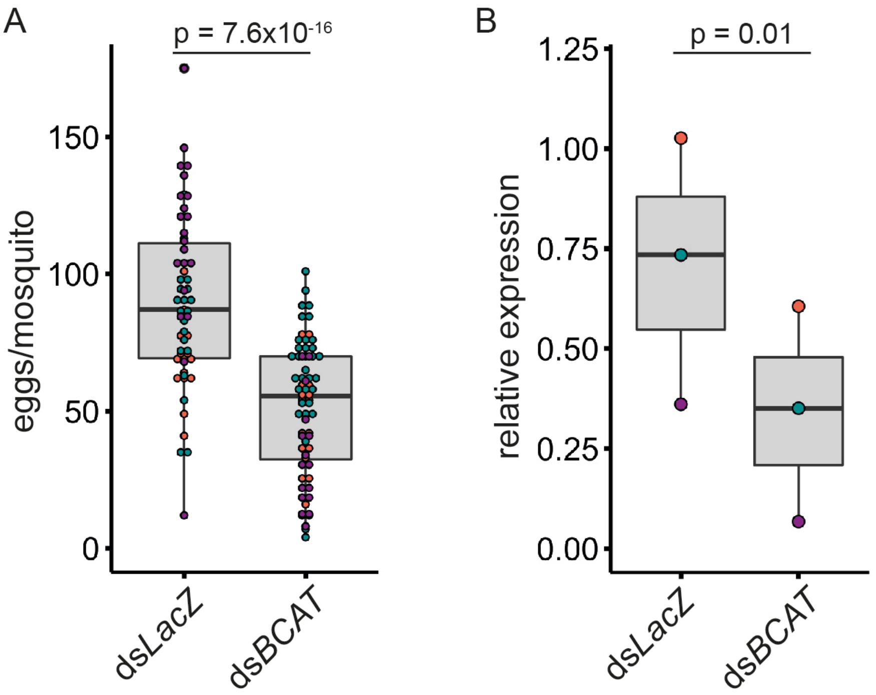

Supplementary Figure 6: Knockdown of *branched chain amino acid transferase* (*BCAT*) reduces mosquito egg production. (A) The number of oviposited eggs per mosquito injected with dsRNA targeting *LacZ* or *BCAT*. Boxplots show the median with first and third quartile, whiskers depict min and max values. Each dot represents number of eggs deposited by one individual female. Significant differences were examined by one-way ANOVA (n>16, N=3) are shown (n = number of mosquitoes within the experiment; N = number of independent experiments). (B) *BCAT* transcript levels normalized to RPS7 in mosquitoes injected with dsRNA targeting *LacZ* or *BCAT*. The color of the dots represents the according experiment in (A). Significant differences examined by one-way ANOVA.

Supplementary Table 1: Primers used in this study.

AGAP number	Abbreviation	Annotation	Forward (5‘ – 3‘)	Reverse (5‘ – 3‘)
AGAP000011	*BCAT*	Branched chain amino acid transferase 2	*GGGACGGCTACTGGAACTGTT*	*GGATCTCCTCACCCATGTACGA*
AGAP000011	*BCAT (3'UTR)*	Branched chain amino acid transferase 2	*ATTCTAGGCGATCGCAGTGAGTTAGCGTGGGTGTCAC*	*TTTATTGCGGCCAGCAGATGCGGATGCGAAAGGATAG*
AGAP012081/ AGAP012106	*ATPase*	ATP synthase 1	*ACCCCAGAAAAGCATCCAAC*	*CGGTGGTCATGAGCGATTTCA*
AGAP004437	*GPD*	Glycerol-3-phosphate dehydrogenase	*TTTGCGACTGTCGTTCCTGA*	*CTTCTCCTTCAGTGTGCGGT*
AGAP002499	*MMSDH*	Methylmalonate-semialdehyde dehydrogenase	*GACGCGTGTCCCCAAATG*	*TGTCTGCTCAGGATGGACGAT*
AGAP010228	*MCC*	3-methylcrotonyl-CoA carboxylase	*GATGAGAACGGCATGATTGGT*	*GCAGTTACACTGGATGCATCGA*
AGAP010464	*NADH (I)*	NADH dehydrogenase	*TCGAACAAAGGACGAAGCGA*	*AATGCGCCGACGTATACCAT*
AGAP003136	*BCKDH*	Branched-chain alpha-keto acid dehydrogenase	*GACCTTATCGTCCCTGTCAACAG*	*GGGCTACGTCTTGCCAAAAC*
AGAP000448	*MOR*	Mitochondrial ornithine receptor	*GCATCAATCAGCAGATCTGAACA*	*GCACGCTGCGGTCCTTA*
AGAP010592	*RPS7*	Ribosomal protein S7	*CATCGAACACAAAGTTGACAC*	*CTAGCACGCAACCCTTATATT*
AGAP013592	miR-276-5p	miR-276-5p	Qiagen Primer	*GAATCGAGCACCAGTTACGC*

Supplementary Table 2: Antagomir list

Name	Sequence
anti-miR-276-5p	5'-*U.*.A.*.G.G.A.A.C.U.C.U.A.U.A.C.C.U.C.*.G.*.C.*.U.*.A*.mN.3'-Chl-3'
scramble	5'-*A.*.A.*.U.G.G.A.C.C.C.U.A.C.U.A.A.U.U.*.U.*.G.*.C.*.C*.mN.3'-Chl-3'

* = phosphothioate backbone; mN = OCH_3-group; Chl = Cholesterol

Supplementary Table 3: Sample sizes in infection and fertility experiments

Experiment	Replicate	Unit (n)	n per experiment
Egg laying rate: miR-276 knockdown	1	mosquito	12 (scramble);<br>9 (miR-276^{KD})
	2	mosquito	13 (scramble);<br>14 (miR-276^{KD})
	3	mosquito	17 (scramble);<br>16 (miR-276^{KD})
Egg laying rate: *BCAT* knockdown	1	mosquito	21 (ds*LacZ*)<br>33 (ds*BCAT*)
	2	mosquito	15 (ds*LacZ*)<br>18 (ds*BCAT*)
	3	mosquito	20 (ds*LacZ*)<br>15 (ds*BCAT*)
Oocyst number	1	midgut	25 (scramble);<br>28 (miR-276^{KD})
	2	midgut	16 (scramble);<br>17 (miR-276^{KD})
	3	midgut	21 (scramble);<br>20 (miR-276^{KD})
Oocyst size	1	oocyst	191 (scramble);<br>87 (miR-276^{KD})
	2	oocyst	117 (scramble);<br>131 (miR-276^{KD})
	3	oocyst	85 (scramble);<br>70 (miR-276^{KD})
Sporozoites number	1	mosquito salivary gland	30 (scramble); pooled<br>30 (miR-276^{KD}); pooled
	2	mosquito salivary gland	30 (scramble); pooled<br>30 (miR-276^{KD}); pooled
	3	mosquito salivary gland	30 (scramble); pooled<br>30 (miR-276^{KD}); pooled
