# Supplementary material for: Metabolic balancing by miR-276 shapes the mosquito reproductive cycle and Plasmodium falciparum development
Source: Nat Commun. 2019 Dec 10;10:5634. doi: 10.1038/s41467-019-13627-y (PMC6904670; doi:10.1038/s41467-019-13627-y)
Supplement: Supplementary file 3 — Reporting Summary [file 41467_2019_13627_MOESM3_ESM.pdf]

nature research

Corresponding author(s): Elena Levashina

Last updated by author(s): Nov 4, 2019

Reporting Summary

Nature Research wishes to improve the reproducibility of the work that we publish. This form provides structure for consistency and transparency in reporting. For further information on Nature Research policies, see Authors & Referees and the Editorial Policy Checklist .

Statistics

For all statistical analyses, confirm that the following items are present in the figure legend, table legend, main text, or Methods section.

n/a	Confirmed	
☐	☒	The exact sample size (*n*) for each experimental group/condition, given as a discrete number and unit of measurement
☐	☒	A statement on whether measurements were taken from distinct samples or whether the same sample was measured repeatedly
☐	☒	The statistical test(s) used AND whether they are one- or two-sided<br>*Only common tests should be described solely by name; describe more complex techniques in the Methods section.*
☐	☒	A description of all covariates tested
☐	☒	A description of any assumptions or corrections, such as tests of normality and adjustment for multiple comparisons
☐	☒	A full description of the statistical parameters including central tendency (e.g. means) or other basic estimates (e.g. regression coefficient) AND variation (e.g. standard deviation) or associated estimates of uncertainty (e.g. confidence intervals)
☐	☒	For null hypothesis testing, the test statistic (e.g. *F*, *t*, *r*) with confidence intervals, effect sizes, degrees of freedom and *P* value noted<br>*Give P values as exact values whenever suitable.*
☒	☐	For Bayesian analysis, information on the choice of priors and Markov chain Monte Carlo settings
☒	☐	For hierarchical and complex designs, identification of the appropriate level for tests and full reporting of outcomes
☒	☐	Estimates of effect sizes (e.g. Cohen's *d*, Pearson's *r*), indicating how they were calculated

Our web collection on statistics for biologists contains articles on many of the points above.

Software and code

Policy information about availability of computer code

Data collection	NA
Data analysis	NA

For manuscripts utilizing custom algorithms or software that are central to the research but not yet described in published literature, software must be made available to editors/reviewers. We strongly encourage code deposition in a community repository (e.g. GitHub). See the Nature Research guidelines for submitting code & software for further information.

Data

Policy information about availability of data

All manuscripts must include a data availability statement. This statement should provide the following information, where applicable:

- Accession codes, unique identifiers, or web links for publicly available datasets
- A list of figures that have associated raw data
- A description of any restrictions on data availability

The polar and lipid LC-MS data in positive and negative polarity as well as GC-MS data that support the findings of this study have been deposited in the MetaboLights repository with the accession code MTBLS1282 [http://www.ebi.ac.uk/metabolights/MTBLS1282]. The manuscript is available as preprint on the bioRxiv server [https://www.biorxiv.org/content/10.1101/548784v1]. Source data for figures 1, 2, 4 and Supplementary Figures 1-6 are provided.

Field-specific reporting

Please select the one below that is the best fit for your research. If you are not sure, read the appropriate sections before making your selection.

Life sciences study design

All studies must disclose on these points even when the disclosure is negative.

Sample size	Experiments were performed in at least three independent repeats accounting for normal biological distribution and therefore allowing statistical analysis using ANOVA and t-test. The supplementary table 3 includes all sample sizes for infection and fertility experiments. Furthermore, each figure legend describes the number of technical and biological replicates.
Data exclusions	All data was included.
Replication	The experiments were repeated at least three times and all results are presented in the manuscript. Each figure legend includes the number of technical and independent repeats. For infection and egg laying experiments the number of mosquitoes and independent repeats is described in Supplementary Table 3. The metabolomics study included six independent replicates.
Randomization	Samples were randomized for mass spectrometry measurements. Furthermore, mosquitoes were co-housed in the same cage using different compartments allowing simultaneous blood feeding (infectious and normal) from the same blood source to avoid housing and/or blood source bias.
Blinding	The investigators were not blinded for experiments comprising objective measurements such as qPCR, metabolomics, ELISA and luciferase activity. In these cases, the analysis should not be influenced by a potential investigators bias.<br>Furthermore, investigators were blinded for analysis of the sporozoite number per mosquito, but not oocyst number per mosquito. However, the latter was performed by two different investigators coming to the same conclusion.

Reporting for specific materials, systems and methods

We require information from authors about some types of materials, experimental systems and methods used in many studies. Here, indicate whether each material, system or method listed is relevant to your study. If you are not sure if a list item applies to your research, read the appropriate section before selecting a response.

Materials & experimental systems

n/a	Involved in the study	
☒	☐	Antibodies
☐	☒	Eukaryotic cell lines
☒	☐	Palaeontology
☐	☒	Animals and other organisms
☒	☐	Human research participants
☒	☐	Clinical data

Methods

n/a	Involved in the study	
☒	☐	ChIP-seq
☒	☐	Flow cytometry
☒	☐	MRI-based neuroimaging

Eukaryotic cell lines

Policy information about cell lines

Cell line source(s)	The Drosophila Schneider S2R+ line was obtained from Drosophila Genomics Resource Center (DGRC) stock number 150.
Authentication	The Drosophila S2R+ line was not additionally authenticated.
Mycoplasma contamination	The cells were not tested for mycoplasma contamination.
Commonly misidentified lines (See ICLAC register)	NA

Animals and other organisms

Policy information about studies involving animals; ARRIVE guidelines recommended for reporting animal research

Laboratory animals	The mosquito strain Anopheles coluzzii Ngousso (TEP1*S1) strain was used throughout the study. For parasite infections, the Plasmodium strain NF54 was used that was a kind gift from Prof. R. Sauerwein, RUMC, The Netherlands.
Wild animals	*Provide details on animals observed in or captured in the field; report species, sex and age where possible. Describe how animals were caught and transported and what happened to captive animals after the study (if killed, explain why and describe method; if released, say where and when) OR state that the study did not involve wild animals.*
Field-collected samples	*For laboratory work with field-collected samples, describe all relevant parameters such as housing, maintenance, temperature, photoperiod and end-of-experiment protocol OR state that the study did not involve samples collected from the field.*

Ethics oversight	No ethical approval was required to perform the experiments on mosquitoes.

Note that full information on the approval of the study protocol must also be provided in the manuscript.
