# Supplementary material for: Metabolic balancing by miR-276 shapes the mosquito reproductive cycle and Plasmodium falciparum development
Source: Nat Commun. 2019 Dec 10;10:5634. doi: 10.1038/s41467-019-13627-y (PMC6904670; doi:10.1038/s41467-019-13627-y)
Supplement: Supplementary file 4 — Description of Additional Supplementary Files [file 41467_2019_13627_MOESM4_ESM.pdf]

Description of Additional Supplementary Files

File Name: Supplementary Data 1
Description: miRNA target prediction. The miRANDA, RNAhybrid and MicroTar algorithms were used to predict miR-276-5p targets.

File Name: Supplementary Data 2
Description: Metabolites that were included in the heatmap. The table depicts the metabolite annotations for the feature, the F- and Bonferroni-corrected p-value of the two-way ANOVA analysis, and the heatmap cluster annotation shown in Figure 3A.

File Name: Supplementary Data 3
Description: Unprocessed metabolite quantification data from LC- and GC-MS platform.
